# Race, Ethnic, and Sex Differences in Prevalence of and Trends in Hallucinogen Consumption Among Lifetime Users in the United States Between 2015 and 2019

**DOI:** 10.3389/fepid.2022.876706

**Published:** 2022-03-23

**Authors:** Alan K. Davis, Brooke J. Arterberry, Yitong Xin, Gabrielle Agin-Liebes, Corrine Schwarting, Monnica T. Williams

**Affiliations:** ^1^Center for Psychedelic Drug Research and Education, College of Social Work, The Ohio State University, Columbus, OH, United States; ^2^Department of Psychiatry and Behavioral Sciences, Center for Psychedelic and Consciousness Research, Johns Hopkins University, Baltimore, MD, United States; ^3^Center for Studies of Drugs, Alcohol, Smoking, and Health (DASH Center), University of Michigan, Ann Arbor, MI, United States; ^4^Department of Psychiatry, University of California, San Francisco, San Francisco, CA, United States; ^5^Psychology Department, Iowa State University, Ames, IA, United States; ^6^School of Psychology, University of Ottawa, Ottawa, ON, Canada

**Keywords:** hallucinogen, epidemiology, NSDUH, race, ethnicity, sex

## Abstract

**Background:**

The current study is one of the first to examine race, ethnic, and sex differences in the prevalence of and trends in hallucinogen use among lifetime users in the United States.

**Methods:**

Data came from the 2015–2019 National Survey on Drug Use and Health and included respondent's reporting ever-using hallucinogens (*n* = 41,060; female = 40.4%). Descriptive and multinomial logistic regression analyses were conducted in Stata.

**Results:**

Highest prevalence of past year hallucinogen use was among Asian females (35.06%), which was two-or-more times larger than prevalence of past year use among White males/females and Native American males. More than half of White males/females, Multiracial males, and Hispanic males reported had ever-used psilocybin or LSD, whereas less than one-quarter of Black males/females reported lifetime psilocybin use, and less than a third of Black females reported lifetime LSD use. Native American males had the lowest prevalence of lifetime MDMA use (17.62–33.30%) but had the highest lifetime prevalence of peyote use (40.37–53.24%). Pacific Islander males had the highest prevalence of lifetime mescaline use (28.27%), and lifetime DMT use was highest among Pacific Islander males/females (15.68–38.58%). Black, Asian, and Multiracial people had greater odds of past-year (ORs = 1.20–2.02; *p*s < 0.05) and past-month (ORs = 1.39–2.06; *p*s < 0.05) hallucinogen use compared to White people. Females had lower odds of past-year (OR = 0.79; *p*s < 0.05), past-month (OR = 0.78; *p*s < 0.05) hallucinogen use compared to males, except for lifetime use of MDMA (OR = 1.29; *p*s < 0.05).

**Conclusions:**

These findings should inform public health initiatives regarding potential benefits and risks of hallucinogen use among racial/ethnic groups and women.

## Introduction

Hallucinogens are powerful psychoactive substances (e.g., d-lysergic acid diethylamide [LSD], psilocybin [“magic mushrooms”], Peyote [Mescaline], Dimethyltryptamine [DMT], and Ayahuasca (1)) that produce shifts in perception, mood, and cognition (2). Hallucinogen use declined from the 1970–1980's but has increased recently, particularly among high school and college students (3). For example, according to the 2018 National Survey on Drug Use and Health (NSDUH), 15.9% of people aged 12 or older, 16.2% of people aged 18–25, and 22.7% of people aged 26–34, are identified as lifetime users (4). One important reason for its popularity could be because hallucinogens are considered relatively safe with little or no risk for dependence (2).

Although hallucinogen use shares a rich history of religious/mystical experiences in almost all cultures, there is a dearth of published information regarding prevalence of and trends in use among Black, Indigenous, and People of Color (BIPOC) and different sex groups in the United States (US). Indeed, many women and BIPOC have been underrepresented in almost every scientific publication on the topic of hallucinogens, possibly due to a number of factors, including social stigma regarding hallucinogen use, racial discrimination, and fear of consequences with reporting use of an illegal drug. For example, people of color are more likely to be charged for crimes at a higher rate compared to Non-Hispanic Whites (5–7). Consistent with this hypothesis, Jahn et al.'s findings (4) showed that BIPOC generally report hallucinogen use at lower rates than Non-Hispanic Whites, possibly due to higher criminalization of BIPOC communities. Similarly, other studies have shown that hallucinogen use was reported significantly more by non-Hispanic White individuals and the fewest reports were from African Americans (8). Regarding sex differences, women tend to report earlier first opportunity to use hallucinogens (9), but hallucinogen use is mostly reported by males (8, 10).

Few studies have provided useful information about the scope of hallucinogen use among BIPOC and female populations and no study has explored whether there are differences in the prevalence of and trends in hallucinogen use between different BIPOC groups as a function of sex. Therefore, the present epidemiological study explores the prevalence of and trends in hallucinogen use among lifetime users in the US as a function of BIPOC and female sex identities.

## Methods

This study used data collected annually from the 2015–2019 National Survey of Drug Use and Health (NSDUH). The NSDUH is a cross-sectional, one-time, survey that uses a stratified independent, multi-stage area probability sampling design to obtain annual data from cohorts of non-institutionalized individuals ages 12 years and older in each state in the United States. Therefore, the NSDUH provides representative population estimates for a variety of substance use-related behaviors in the general United States population. Households are selected with in-person screening to identify residents aged 12 and older, with full interviews conducted on a random sample of household members. The NSDUH includes audio computer-assisted self-interviewing to ensure privacy, accuracy, and completeness and to try and reduce sub-notification. Response rates for the NSDUH are reliably above 80% for the screening sample and 70% for the full weighted interview samples. Further details regarding NSDUH methodology are reported elsewhere (11, 12). This study was determined exempt by the Iowa State University Institutional Review Board.

### Measures

Lifetime, past-year, and past-month hallucinogen use was assessed. Specific hallucinogens were assessed for lifetime use and included peyote, mescaline, psilocybin, Lysergic acid diethylamide (LSD), 3,4-methylenedioxymethamphetamine (MDMA), and N-dimethyltryptamine (DMT). Past year alcohol, marijuana, and tobacco use was assessed. Sociodemographic and other control variables included biological sex, race/ethnicity, age, highest educational status, income, and population density.

### Statistical Analysis

All analyses used Stata, version 16.0 (StataCorp, LLC), using the *svy* and *margins* commands accounting for complex survey design. Additionally, the NSDUH data were weighted and clustered on primary sampling units. Taylor series approximations with adjusted degrees of freedom were used to maintain robust variance. Potential change of hallucinogen use over the course of the NSDUH years was evaluated using a series of logistic regressions for past year hallucinogen use, past month hallucinogen use, and lifetime use for the following hallucinogens: peyote, mescaline, psilocybin, LSD, MDMA, and DMT. Given their clinical importance to understanding lifetime use of hallucinogens, predictors included several geographic (e.g., population density), demographic (e.g., age, sex income), and concomitant substance use (e.g., alcohol, tobacco) variables. Estimates of linearized annual change *via* the Stata *margins* command was used to calculate potential trend slope and adjust for average covariate values within the study period. The *subpop* option was used for analyses (e.g., lifetime hallucinogen use). All analyses used a statistical significance 2-sided *P* < 0.05. For prevalence of hallucinogen use across race/ethnicity and biological sex, we used weighted cross-tabulations. The *margins* command evaluated annual change in models associated with year while controlling for age, past year alcohol use, past year tobacco use, past year marijuana use, highest level of education, income, and population density. Odds ratios with standard error and a 95% confidence interval were reported.

## Results

Between 2015 and 2019, the weighted sample of respondents were aged 12 and older and reported lifetime use of hallucinogens (*n* = 41,060). The weighted sample was male (59.6 %) and White (76.6 %). Approximately 35.8% of respondents were aged 50 or older, 31.8% reported being a college graduate, 15.9% reported having an income of $50,000–74,999, and 55.6% reported living in a segment in a CBSA with 1 million people or more persons.

### Prevalence Estimates of Hallucinogen Use

As Table 1 shows, from 2015 to 2019, among those that reported ever using hallucinogens, most respondents reported lifetime psilocybin, LSD, or MDMA use compared to mescaline or DMT (see Table 1). Specifically, 50% or more of White males/females, Multiracial males, and Hispanic males had ever used psilocybin or LSD, whereas less than one-quarter of Black males/females reported lifetime psilocybin use, and less than a third of Black females reported lifetime LSD use. Additionally, Native American males had the lowest prevalence of lifetime MDMA use (17.62% [2018]-33.30% [2017]). The lifetime prevalence of peyote use was highest among Native American males (40.37% [2015]-53.24% [2017]) and females (31.92% [2017]-51.15% [2016]). Pacific Islander males had the highest prevalence of lifetime mescaline use (28.27% [2017]), followed by White males (26.55% [2016]), and Multiracial males (26.51% [2019]). Lifetime DMT use was highest among Pacific Islander males/females (15.68% [2015]-38.58% [2017]), with 6% or less of White females, Black males/females, and Native American and Asian females reporting lifetime DMT use.

**Table 1:** Percentage of hallucinogen use among those that ever used hallucinogens by race/ethnicity and biological sex 2015–2019.

**Race/ethnicity**	**Sex**	**Year**	**Unweighted sample size**	**Past year Hallucinogen use**	**Past month Hallucinogen use**	**Ever used Peyote**	**Ever used Mescaline**	**Ever used Psilocybin**	**Ever used LSD**	**Ever used MDMA**	**Ever used DMT**
White	Male	2015	3,297	11.31	2.96	17.84	24.56	66.39	70.73	40.77	7.64
White	Male	2016	3,264	11.00	2.61	18.65	26.55	65.68	70.73	40.31	6.37
White	Male	2017	3,395	11.68	3.38	17.87	22.15	69.63	70.52	41.09	7.71
White	Male	2018	3,356	11.58	3.15	18.52	21.63	70.73	70.24	40.73	8.05
White	Male	2019	3,250	12.61	4.08	16.83	22.63	67.35	70.82	40.26	8.84
White	Female	2015	2,660	7.36	1.51	11.21	18.53	52.28	61.74	42.45	3.45
White	Female	2016	2,561	9.58	2.17	9.55	14.78	50.50	62.22	44.28	4.06
White	Female	2017	2,515	8.94	2.90	9.90	17.87	51.27	62.93	45.09	4.70
White	Female	2018	2,489	10.80	2.59	10.03	15.50	52.26	61.39	47.69	5.07
White	Female	2019	2,529	10.02	3.04	9.15	14.12	53.30	61.86	44.88	5.41
Black	Male	2015	316	20.36	7.62	4.96	9.53	19.41	32.04	59.39	3.25
Black	Male	2016	316	14.91	3.58	5.82	21.99	23.22	45.76	45.73	2.57
Black	Male	2017	306	14.25	4.04	5.35	14.99	27.06	38.67	47.81	4.41
Black	Male	2018	295	23.15	11.11	6.63	14.36	29.33	47.99	51.67	2.54
Black	Male	2019	285	20.40	5.64	6.47	13.74	32.35	40.10	50.27	11.30
Black	Female	2015	235	13.81	4.15	1.01	8.19	9.24	23.54	67.07	1.26
Black	Female	2016	229	13.68	4.59	2.38	8.38	11.96	30.35	55.01	2.38
Black	Female	2017	205	22.06	5.14	0.66	5.93	9.61	18.60	64.47	2.36
Black	Female	2018	212	13.66	3.12	0.75	8.91	9.21	29.26	66.26	0.71
Black	Female	2019	212	16.05	6.90	4.28	7.40	22.99	35.95	62.93	4.26
Native American	Male	2015	114	7.23	1.37	40.37	6.83	51.61	45.04	32.50	8.91
Native American	Male	2016	92	13.61	5.66	49.69	21.81	43.21	49.79	23.57	2.32
Native American	Male	2017	86	7.18	0.00	53.24	9.69	47.76	59.27	33.30	7.86
Native American	Male	2018	72	8.48	1.72	51.71	8.79	43.23	48.12	17.62	4.66
Native American	Male	2019	90	21.81	14.00	41.99	9.41	48.78	36.35	32.93	3.30
Native American	Female	2015	76	6.82	5.31	39.91	1.82	47.11	38.30	40.79	3.67
Native American	Female	2016	77	17.65	7.10	51.15	11.40	32.27	35.74	24.98	1.38
Native American	Female	2017	76	14.96	3.09	31.92	1.09	38.49	49.39	46.06	6.79
Native American	Female	2018	68	13.20	2.45	33.52	7.23	40.33	41.87	43.56	4.51
Native American	Female	2019	74	13.30	3.32	46.52	1.94	41.55	31.52	30.46	3.53
Hawaiian/Pacific Islander	Male	2015	13	11.40	3.43	11.20	5.04	44.03	14.23	70.99	0.00
Hawaiian/Pacific Islander	Male	2016	22	17.27	0.00	6.94	0.00	59.09	45.42	62.46	6.94
Hawaiian/Pacific Islander	Male	2017	11	29.18	9.69	16.81	28.27	48.08	48.41	41.00	36.06
Hawaiian/Pacific Islander	Male	2018	24	3.98	0.45	7.72	0.00	21.59	54.39	55.60	6.01
Hawaiian/Pacific Islander	Male	2019	20	10.14	0.00	11.97	0.00	30.57	50.87	73.97	0.66
Hawaiian/Pacific Islander	Female	2015	12	44.72	16.36	2.37	0.00	83.70	89.66	68.10	15.68
Hawaiian/Pacific Islander	Female	2016	18	1.75	1.75	0.70	0.70	19.56	27.93	64.96	1.50
Hawaiian/Pacific Islander	Female	2017	10	45.88	27.92	29.88	0.00	58.01	74.00	35.33	38.58
Hawaiian/Pacific Islander	Female	2018	11	15.41	0.00	0.37	0.37	81.85	77.84	44.41	0.37
Hawaiian/Pacific Islander	Female	2019	17	33.48	1.13	8.04	0.00	32.60	46.08	71.75	3.72
Asian	Male	2015	93	25.37	6.39	0.49	1.40	31.90	35.86	77.95	7.21
Asian	Male	2016	85	25.30	8.39	15.08	6.37	60.41	43.49	56.03	4.63
Asian	Male	2017	89	22.47	2.37	3.87	7.82	59.01	51.28	67.34	12.56
Asian	Male	2018	106	23.27	10.48	3.59	4.02	52.77	37.67	63.49	4.41
Asian	Male	2019	115	27.41	12.34	1.74	2.71	51.46	41.75	63.36	10.86
Asian	Female	2015	95	35.06	4.88	9.64	9.43	35.94	33.34	66.57	0.24
Asian	Female	2016	74	19.17	8.44	1.21	0.61	47.60	44.04	76.00	1.20
Asian	Female	2017	70	27.17	9.58	0.00	0.24	37.57	34.94	68.50	2.92
Asian	Female	2018	89	20.00	4.69	1.32	8.54	34.05	49.61	49.34	3.45
Asian	Female	2019	84	23.03	12.90	4.81	2.22	31.62	37.39	69.23	6.10
Multiracial	Male	2015	205	19.00	9.04	17.78	21.22	60.93	70.90	48.22	5.68
Multiracial	Male	2016	212	16.42	3.14	19.28	18.48	62.67	58.36	47.06	6.56
Multiracial	Male	2017	222	19.93	3.99	16.04	18.13	65.21	50.88	50.11	8.07
Multiracial	Male	2018	230	19.80	9.52	20.07	19.15	59.88	69.47	45.96	6.51
Multiracial	Male	2019	219	15.07	4.69	22.55	26.51	60.39	71.03	41.58	13.73
Multiracial	Female	2015	217	17.31	4.13	10.25	21.14	53.48	58.51	50.71	3.84
Multiracial	Female	2016	175	16.31	8.01	17.78	19.20	53.08	54.62	61.86	7.67
Multiracial	Female	2017	184	14.56	3.61	5.10	21.62	35.05	52.99	48.43	5.03
Multiracial	Female	2018	185	16.05	4.97	20.18	11.49	47.70	55.80	36.46	8.69
Multiracial	Female	2019	200	17.91	4.96	8.73	17.49	46.63	52.58	53.62	11.34
Hispanic	Male	2015	629	16.73	4.75	10.62	14.03	52.10	54.34	53.98	4.69
Hispanic	Male	2016	523	18.22	7.73	10.17	12.73	49.37	57.14	51.11	9.38
Hispanic	Male	2017	570	17.29	4.95	11.24	9.84	49.22	53.33	57.49	6.16
Hispanic	Male	2018	579	15.15	5.20	13.02	12.01	57.43	61.88	59.25	7.23
Hispanic	Male	2019	628	21.48	6.31	13.47	11.64	48.65	53.24	52.81	9.12
Hispanic	Female	2015	492	20.40	4.54	2.53	7.69	34.90	45.51	63.24	4.96
Hispanic	Female	2016	435	16.41	5.30	8.92	15.82	42.44	47.61	56.50	4.69
Hispanic	Female	2017	433	15.01	4.45	4.42	6.68	44.27	44.35	57.35	2.85
Hispanic	Female	2018	438	15.53	4.41	12.98	12.51	36.93	47.83	60.70	7.96
Hispanic	Female	2019	474	17.17	4.26	4.84	5.10	37.64	51.82	63.97	5.97

*Ever used hallucinogens unweighted sample size = 41,060; Ever used, Past-month, and Past-year weighted percentages based on having ever used hallucinogens during lifetime*.

Between 2015 and 2019, there was an annual increase in past year hallucinogen use, past month hallucinogen use, ever use of MDMA, and ever use of DMT across race/ethnicity and biological sex (see Table 2). There was an annual decrease in lifetime use of peyote and mescaline. No observed annual changes were found for lifetime use of psilocybin or LSD. Notably, there was no annual increase/decrease of lifetime peyote or mescaline use among Pacific Islander males and females, nor annual increase/decrease of lifetime peyote use among Asian males or females.

**Table 2:** Annualized change percentages from 2015 to 2019, by race/ethnicity and biological sex.

	**Past year Hallucinogen use (%, 95% CI)**	**Past month Hallucinogen use (%, 95% CI)**	**Ever used Peyote (%, 95% CI)**	**Ever used Mescaline (%, 95% CI)**	**Ever used Psilocybin** **(%, 95% CI)**	**Ever used LSD (%, 95% CI)**	**Ever used MDMA** **(%, 95% CI)**	**Ever used DMT (%, 95% CI)**
Full sample	**0.50 (0.31, 0.68)**	**0.22 (0.13, 0.33)**	**−0.36 (−0.71,−0.01)**	**−1.09 (−0.16,−0.60)**	0.06 (−0.47, 0.60)	−0.04 (−0.52, 0.44)	**0.39 (0.06, 0.72)**	**0.40 (0.06, 0.72)**
White, Male	**0.71 (0.45, 0.97)**	**0.22 (0.12, 0.31)**	**−0.51 (−0.99,−0.03)**	**−1.36 (−1.96,−0.76)**	0.07 (−0.51, 0.64)	−0.04 (−0.50, 0.41)	**0.37 (0.06, 0.68)**	**0.61 (0.37, 0.84)**
White, Female	**0.38 (0.24, 0.52)**	**0.15 (0.08, 0.22)**	**−0.36 (−0.70,−0.01)**	**−1.19 (−1.73−0.06)**	0.07 (−0.53, 0.68)	−0.04 (−0.53 0.45)	**0.38 (0.06, 0.67)**	**0.37 (0.23, 0.50)**
Black, Male	**0.71 (0.46, 0.97)**	**0.40 (0.20, 0.61)**	**−0.17 (−0.34,−0.002)**	**−0.88 (−0.13,−0.47)**	0.04 (−0.32, 0.41)	−0.04 (−0.52, 0.43)	**0.42 (0.07, 0.77)**	**0.40 (0.16, 0.63)**
Black, Female	**0.55 (0.35, 0.75)**	**0.27 (0.13, 0.41)**	**−0.10 (−0.21, 0.003)**	**−0.73 (−1.09,−0.37)**	0.03 (−0.20, 0.26)	−0.04 (−0.48, 0.40)	**0.41 (0.07, 0.76)**	**0.22 (0.09, 0.36)**
Native American, Male	**0.62 (0.37, 0.87)**	**0.36 (0.09, 0.64)**	−0.79 (−1.52, 0.04)	**−0.79 (−0.12,−0.36)**	0.07 (−0.51, 0.65)	−0.04 (−0.56, 0.47)	**0.32 (0.49, 0.60)**	**0.48 (0.23, 0.73)**
Native American, Female	**0.46 (0.27, 0.65)**	**0.25 (0.06, 0.44)**	**−0.75 (−0.50, 0.08)**	**−0.73 (−1.15,−0.31)**	0.06 (−0.43, 0.55)	−0.05 (−0.54, 0.45)	**0.33 (0.05, 0.61)**	**0.29 (0.14, 0.44)**
Hawaiian/Pacific Islander, Male	**0.56 (0.30, 0.83)**	**0.18 (0.06, 0.29)**	−0.33 (−1.45, 0.04)	−0.32 (−0.72, 0.08)	0.07 (−0.51, 0.64)	−0.05 (−0.57, 0.47)	**0.42 (0.07, 0.77)**	**0.66 (0.25, 1.08)**
Hawaiian/Pacific Islander, Female	**0.43 (0.22, 0.62)**	**0.13 (0.05, 0.21)**	−0.21 (−0.50, 0.08)	−0.23 (−0.54, 0.07)	0.06 (−0.42, 0.53)	−0.05 (−0.56, 0.47)	**0.42 (0.07, 0.78)**	**0.43 (0.14, 0.71)**
Asian, Male	**0.73 (0.47, 0.99)**	**0.38 (0.20, 0.55)**	−0.25 (−0.52, 0.03)	**−0.59 (−1.00,−0.18)**	0.07 (−0.52, 0.66)	−0.05 (−0.57, 0.47)	**0.43 (0.07, 0.79)**	**0.49 (0.26, 0.73)**
Asian, Female	**0.58 (0.37, 0.79)**	**0.28 (0.14, 0.39)**	−0.15 (−0.33, 0.03)	**−0.47 (−0.82,−0.12)**	0.06 (−0.43, 0.55)	−0.05 (−0.54, 0.45)	**0.43 (0.07, 0.79)**	**0.30 (0.15, 0.45)**
Multiracial, Male	**0.83 (0.53, 1.14)**	**0.46 (0.26, 0.68)**	**−0.48 (−0.93,−0.02)**	**−1.13 (−1.66,−0.60)**	0.07 (−0.51, 0.65)	−0.45 (−0.54, 0.45)	**0.40 (0.06, 0.74)**	**0.82 (0.47, 1.17)**
Multiracial, Female	**0.67 (0.42, 0.91)**	**0.34 (0.19, 0.49)**	**−0.36 (−0.72,−0.01)**	**−1.05 (−1.55,−0.55)**	0.07 (−0.49, 0.62)	−0.05 (−0.56, 0.46)	**0.40 (0.06, 0.73)**	**0.50 (0.27, 0.71)**
Hispanic, Male	**0.65 (0.41, 0.88)**	**0.33 (0.17, 0.48)**	**−0.37 (−0.73,−0.01)**	**−0.89 (−1.31,−0.48)**	0.07 (−0.51, 0.65)	−0.05(−0.56, 0.47)	**0.43 (0.07, 0.80)**	**0.61 (0.37, 0.85)**
Hispanic, Female	**0.52 (0.34, 0.72)**	**0.24 (0.12, 0.35)**	**−0.26 (−0.51,−0.006)**	**−0.76 (−1.01,−0.39)**	0.06 (−0.42, 0.54)	−0.05 (−0.56, 0.47)	**0.43 (0.07, 0.80)**	**0.37 (0.22, 0.53)**

*Ever used hallucinogens unweighted sample size = 41,060; Bold = p <0.05*.

### Predictors of Hallucinogen Use in Multinomial Logistic Regression Models

As Table 3 shows, those identifying as Black, Asian, and Multiracial had greater odds of past-year (ORs = 1.20–2.02; *p*s < 0.05) and past-month (ORs = 1.39–2.06; *p*s < 0.05) hallucinogen use when compared to white respondents. Additionally, compared to white respondents, those identifying as Black, Asian, and Hispanic had greater odds (ORs = 1.27–1.65; *p*s < 0.05) of lifetime MDMA use, and Native Americans had greater odds (OR = 7.95; *p* <0.05) of lifetime peyote use. Compared to males, females had lower odds of past-year (OR = 0.79; *p*s < 0.05) and past-month (OR = 0.78; *p* < 0.05) hallucinogen use, except for lifetime use of MDMA (OR = 1.29; *p* < 0.05). Compared to younger people (age 12–17), adults (18+) had lower odds of past year (ORs = 0.01–0.20; *p*s < 0.05) and past month (ORs = 0.04–0.50; *p*s < 0.05) hallucinogen use, whereas people aged 26+ had greater odds of lifetime peyote, mescaline, psilocybin, MDMA, or DMT use (ORs = 1.31–23.15; *p*s < 0.05). Conversely, compared to younger people, those 50+ had a lower odds of lifetime MDMA or DMT use (ORs = 0.31–0.35; *p*s < 0.05). People who reported past year alcohol or tobacco use also had greater odds of reporting past year (ORs = 1.28–1.76; *p*s < 0.05) or past month (ORs = 1.36–1.50; *p*s < 0.05) hallucinogen use, as well as lifetime use of MDMA (ORs = 1.05–1.28; *p*s < 0.05). Those who reported past year marijuana use had greater odds of reporting past year (OR = 4.64; *p* < 0.05) or past month (OR = 4.03; *p*s < 0.05) hallucinogen use, as well as lifetime use of all specific hallucinogens (ORs = 1.50–2.30; *p*s < 0.05).

**Table 3:** Race and Sex differences in hallucinogen use 2015–2019.

	**Past year Hallucinogen use** **(*n* = 8,173)**			**Past month Hallucinogen use** **(*n* = 2,230)**			**Ever used Peyote** **(*n* = 3,866)**			**Ever used Mescaline** **(*n* = 4,647)**			**Ever used Psilocybin** **(*n* = 23,032)**			**Ever used LSD** **(*n* = 23,568)**			**Ever used MDMA** **(*n* = 21,962)**			**Ever used DMT** **(*n* = 3,267)**		
	**OR (SE)**	**95% CI**		**OR (SE)**	**95% CI**		**OR (SE)**	**95% CI**		**OR (SE)**	**95% CI**		**OR (SE)**	**95% CI**		**OR (SE)**	**95% CI**		**OR (SE)**	**95% CI**		**OR (SE)**	**95% CI**	
**Race/ethnicity (White-ref)**																								
Black	**1.37 (0.12)**	**1.15**	**1.63**	**1.51 (0.18)**	**1.19**	**1.92**	**0.24 (0.04)**	**0.17**	**0.34**	**0.50 (0.06)**	**0.39**	**0.63**	**0.15 (0.01)**	**0.13**	**0.18**	**0.23 (0.02)**	**0.2**	**0.27**	**1.65 (0.12)**	**1.43**	**1.9**	**0.42 (0.07)**	**0.3**	**0.6**
Native American	0.99 (0.20)	0.65	1.5	1.32 (0.43)	0.69	2.53	**7.95 (1.47)**	**5.49**	**11.52**	**0.43 (0.12)**	**0.25**	**0.74**	**0.51 (0.07)**	**0.39**	**0.69**	**0.34 (0.04)**	**0.27**	**0.43**	**0.49 (0.06)**	**0.38**	**0.63**	**0.53 (0.12)**	**0.34**	**0.82**
Hawaiian/Pacific Islander	0.87 (0.30)	0.43	1.74	0.64 (0.20)	0.34	1.19	0.65 (0.33)	0.24	1.81	**0.13 (0.09)**	**0.03**	**0.55**	**0.50 (0.13)**	**0.29**	**0.84**	0.57 (0.14)	0.35	0.93	**0.17 (0.41)**	**1.06**	**2.78**	0.82 (0.27)	**0.42**	**1.57**
Asian	**2.02 (0.25)**	**1.58**	**2.58**	**2.06 (0.28)**	**1.58**	**2.7**	**0.46 (0.15)**	**0.24**	**0.89**	**0.34 (0.14)**	**0.15**	**0.77**	**0.49 (0.60)**	**0.38**	**0.62**	**0.52 (0.06)**	**0.41**	**0.65**	**1.52 (0.20)**	**1.17**	**1.97**	**0.71 (0.12)**	**0.51**	**1**
Multiracial	**1.20 (0.10)**	**1.02**	**1.42**	**1.39 (0.19)**	**1.06**	**1.82**	**1.27 (0.16)**	**0.99**	**1.63**	1.07 (0.15)	0.8	1.41	**0.78 (0.66)**	**0.66**	**0.93**	**0.73 (0.07)**	**0.61**	**0.88**	1.07 (0.10)	0.89	1.29	0.98 (0.17)	0.76	1.27
Hispanic	1.01 (0.75)	0.87	1.17	1.17 (0.12)	0.94	1.44	0.97 (0.11)	0.77	1.22	**0.74 (0.08)**	**0.6**	**0.91**	**0.57 (0.03)**	**0.51**	**0.64**	**0.64 (0.37)**	**0.57**	**0.72**	**1.27 (0.06)**	**1.15**	**1.39**	**0.72 (0.06)**	**0.6**	**0.86**
**Biological Sex (Male-ref)**																								
Female	**0.79 (0.04)**	**0.72**	**0.86**	**0.78 (0.05)**	**0.7**	**0.91**	**0.56 (0.03)**	**0.45**	**0.57**	**0.64 (0.03)**	**0.58**	**0.71**	**0.53 (0.02)**	**0.49**	**0.56**	**0.73 (0.02)**	**0.69**	**0.77**	**1.29 (0.05)**	**1.2**	**1.39**	**0.59 (0.04)**	**0.51**	**0.68**
**Age (12–17 yrs old-ref)**																								
18–25 yrs old	**0.20 (0.02)**	**0.17**	**0.24**	**0.50 (0.07)**	**0.39**	**0.65**	0.73 (0.12)	0.52	1.02	1.18 (0.23)	0.8	1.73	**1.24 (0.12)**	**1.03**	**1.5**	1.03 (0.84)	0.88	1.22	**2.66 (0.20)**	**2.28**	**3.1**	**1.50 (0.23)**	**1.1**	**2.02**
26–34 yrs old	**0.07 (0.01)**	**0.05**	**0.08**	**0.28 (0.04)**	**0.21**	**0.36**	**1.31 (0.24)**	**0.92**	**1.89**	**2.18 (0.43)**	**1.47**	**3.23**	**1.87 (0.19)**	**1.52**	**2.31**	**0.78 (0.06)**	**0.66**	**0.92**	**3.61 (0.29)**	**3.07**	**4.25**	**1.29 (0.21)**	**0.94**	**1.78**
35–49 yrs old	**0.02 (0.002)**	**0.02**	**0.03**	**0.12 (0.02)**	**0.09**	**0.16**	**2.22 (0.36)**	**1.61**	**3.06**	**4.60 (0.83)**	**3.17**	**6.6**	**2.24 (0.21)**	**1.85**	**2.7**	**2.50 (0.20)**	**2.13**	**2.93**	**1.91 (0.17)**	**1.6**	**2.28**	**0.72 (0.11)**	**0.53**	**0.97**
50 or older	**0.01 (0.001)**	**0.01**	**0.01**	**0.04 (0.01)**	**0.03**	**0.07**	**8.94 (1.54)**	**6.32**	**12.65**	**23.15 (4.33)**	**15.9**	**33.71**	**1.93 (0.18)**	**1.6**	**2.35**	**3.66 (0.33)**	**3.05**	**4.4**	**0.31 (0.03)**	**0.26**	**0.37**	**0.35 (0.06)**	**0.25**	**0.5**
**Past year alcohol use**	**1.76 (0.17)**	**1.44**	**2.15**	**1.36 (0.20)**	**1.01**	**1.84**	**0.71 (0.05)**	**0.62**	**0.82**	**0.68 (0.05)**	**0.59**	**0.79**	**1.00 (0.05)**	**0.91**	**1.11**	**0.74 (0.42)**	**0.66**	**0.83**	**1.05 (0.06)**	**0.93**	**1.17**	**0.63 (0.06)**	**0.53**	**0.7**
**Past year tobacco use**	**1.28 (0.05)**	**1.17**	**1.39**	**1.50 (0.12)**	**1.28**	**1.77**	**0.99 (0.06)**	**0.89**	**1.11**	**0.98 (0.05)**	**0.89**	**1.09**	**1.09 (0.04)**	**1.01**	**1.17**	**1.27 (0.41)**	**1.19**	**1.36**	**1.28 (0.04)**	**1.2**	**1.37**	**1.27 (0.08)**	**1.12**	**1.44**
**Past year marijuana use**	**4.64 (0.27)**	**4.13**	**5.21**	**4.03 (0.47)**	**3.29**	**4.94**	**1.50 (0.08)**	**1.34**	**1.67**	**1.51 (0.08)**	**1.36**	**1.68**	**2.30 (0.08)**	**2.13**	**2.47**	**1.57 (0.05)**	**1.47**	**1.68**	**1.59 (0.0)**	**1.47**	**1.71**	**1.79 (0.09)**	**1.62**	**1.99**
**Highest level of education (less than high school-ref)**																								
High school	**0.88 (0.07)**	**0.75**	**1.04**	**0.08 (0.11)**	**0.61**	**1.05**	**1.09 (0.11)**	**0.89**	**1.34**	**1.10 (0.11)**	**0.9**	**1.34**	**1.22 (0.08)**	**1.07**	**1.1**	**1.02 (0.06)**	**0.9**	**1.15**	**1.10 (0.07)**	**0.97**	**1.26**	**0.96 (0.11)**	**0.77**	**1.21**
Some college/associates degree	**1.10 (0.08)**	**0.95**	**1.27**	**0.80 (0.20)**	**0.63**	**1.01**	**1.36 (0.13)**	**1.12**	**1.64**	**1.31 (0.11)**	**1.1**	**1.56**	**1.53 (0.09)**	**1.35**	**1.73**	**1.00 (0.06)**	**0.89**	**1.11**	**1.18 (0.07)**	**1.04**	**1.34**	**1.10 (0.12)**	**0.88**	**1.37**
College graduate	**1.30 (0.11)**	**1.09**	**1.55**	**1.00 (0.14)**	**0.75**	**1.32**	**1.30 (0.15)**	**1.04**	**1.63**	**0.97 (0.09)**	**0.86**	**1.16**	**1.75 (0.10)**	**1.55**	**1.97**	**9.71 (0.04)**	**0.63**	**0.8**	**1.24 (0.08)**	**1.08**	**1.41**	**0.87 (0.12)**	**0.66**	**1.14**
**Income (< $20,000-ref)**																								
$20,000–49,999	**0.85 (0.05)**	**0.76**	**0.96**	**0.93 (0.08)**	**0.78**	**1.11**	**0.94 (0.09)**	**0.78**	**1.14**	**0.96 (0.08)**	**0.81**	**1.13**	**0.96 (0.04)**	**0.88**	**1.05**	**1.09 (0.05)**	**1**	**1.19**	**1.03 (0.05)**	**0.94**	**1.14**	**0.88 (0.05)**	**0.78**	**1**
$50,000–74,999	**0.70 (0.04)**	**0.2**	**0.78**	**0.82 (0.08)**	**0.68**	**0.98**	**0.80 (0.09)**	**0.64**	**1**	**0.90 (0.08)**	**0.76**	**1.08**	**0.99 (0.05)**	**0.89**	**1.1**	**1.05 (0.05)**	**0.95**	**1.17**	**0.99 (0.06)**	**0.87**	**1.13**	**0.77 (0.06)**	**0.66**	**0.99**
$75,000 or More	**0.67 (0.04)**	**0.58**	**0.76**	**0.72 (0.08)**	**0.57**	**0.9**	**0.75 (0.06)**	**0.63**	**0.89**	**0.81 (0.07)**	**0.8**	**0.97**	**1.00 (0.05)**	**0.91**	**1.1**	**0.89 (0.40)**	**0.81**	**0.97**	**1.05 (0.06)**	**0.94**	**1.18**	**0.59 (0.04)**	**0.51**	**0.69**
**Population density (CBSA> 1 million-ref)**																								
CBSA <1 million persons	**0.85 (0.04)**	**0.77**	**0.94**	**0.86 (0.05)**	**0.76**	**0.96**	1.06 (0.06)	0.95	1.18	0.96 (0.04)	0.88	1.06	1.08 (0.04)	1	1.15	1.02 (0.04)	0.95	1.09	**0.75 (0.02)**	**0.71**	**0.8**	0.95 (0.05)	0.86	1.06
Segment not in a CBSA	**0.64 (0.06)**	**0.53**	**0.77**	**0.63 (0.10)**	**0.46**	**0.85**	1.02 (0.09)	0.84	1.23	0.90 (0.10)	0.72	1.13	1.02 (0.96)	0.85	1.23	1.00 (0.09)	0.83	1.2	**0.63 (0.04)**	**0.55**	**0.72**	0.76 (0.10)	0.56	0.99

*Ever used hallucinogens unweighted sample size = 41,060; Bold p < 0.05; OR, Odds Ratio; SE, Standard Error; CI, Confidence Interval*.

Moreover, in terms of sociodemographic variables, compared to those with less than a high school education, those who graduated college had greater odds of reporting past year (OR = 1.30; *p* < 0.05) and past month (OR = 1.00; *p*s < 0.05) hallucinogen use, as well as lifetime use of peyote (OR = 1.30; *p*s < 0.05), psilocybin (OR = 1.75; *p*s < 0.05), LSD (OR = 9.71; *p*s < 0.05), and MDMA (OR = 1.24; *p*s < 0.05). Lastly, compared to those with income <20,000 dollars per year, those with higher income generally had lower odds of reporting past year (ORs = 0.67–0.85; *p*s < 0.05) or past month (ORs = 0.72–0.93; *p*s < 0.05) hallucinogen use, or reporting lifetime use of any specific hallucinogen (ORs = 0.59–0.99; *p*s < 0.05), except that those reporting income of 75,000 dollars or more had greater odds of lifetime psilocybin (OR = 1.00; *p*s < 0.05) and MDMA (OR = 1.05; *p*s < 0.05) use, those with income between 50,000 and 74,999 dollars had greater odds of LSD (OR = 1.05; *p*s < 0.05) use, and those with income between 20,000 and 49,999 dollars had greater odds of LSD (OR = 1.09; *p*s < 0.05) and MDMA (OR = 1.03; *p*s < 0.05) use.

## Discussion

Among those reporting ever using hallucinogens, most respondents reported lifetime use of psilocybin, LSD, or MDMA. Compared to males, females presented with lower odds of past-month, past-year, and lifetime use of all hallucinogens except MDMA, corroborating previous epidemiological research suggesting disproportionally higher prevalence rates of hallucinogen use among males (10, 13, 14). Notably, people of color reported substantially higher prevalence rates of past-year hallucinogen use than those captured in previous reports (14–17). Furthermore, our findings indicated that from 2015 to 2019 lifetime LSD use remained relatively stable across biological sex and race/ethnicity. Research has consistently demonstrated that hallucinogen use typically occurs among those that identify as White (10, 14). However, the rising prevalence of non-White groups in the present study reflects potential shifting racial/ethnographic trends in use.

Interestingly, Asian-identified males/females reported the highest prevalence of past year hallucinogen use across 2015–2019, which was twice or more as large as the prevalence estimates of White males/females. Additionally, there were twice as many Black and Multiracial males than White, Native American, and Pacific Islander males that reported past month hallucinogen use. Asian Americans/Pacific Islanders have generally reported the lowest aggregate rates of hallucinogen and other drug use in national samples compared to other groups (18). The higher rates suggest that Asian Americans and Pacific Islanders, in addition to Black and Multiracial male individuals, have become more frequent consumers of hallucinogenic substances (18), which is supported by our findings of an average increase in hallucinogen use from 2015 to 2019 among Asian identified males/females. These findings provide preliminary evidence of meaningful shifts in attitudes toward and acceptance of hallucinogens, potentially driven by heightened public interest in these substances for the treatment of several mental health conditions (19) and the possibility of self-medication of the deleterious effects of racial trauma (20).

Perhaps not surprisingly, the lifetime prevalence of peyote use in this study was highest among self-identified Native American males/females. However, there was a decreasing trend regarding peyote use and an increasing trend for DMT and MDMA use among Native American males/females. Even though there was a larger prevalence of peyote use among Native Americans, the trend suggests changes in type of hallucinogens being consumed. Indeed, Native Americans have used peyote cactus as a religious sacrament for millennia (21), and peyote use among members of the Native American Church is legally protected under the American Indian Religious Freedom Act (22). The high prevalence of use likely reflects the steady growth of the Native American population in the US in recent decades, with an increasing number of members joining the Native American Church (22). Given that DMT (in the form of ayahuasca) and MDMA have increasingly been shown to have therapeutic properties [e.g., MDMA: (23); DMT: (24, 25)], it is quite possible that peyote, DMT, and MDMA use are not considered a recreational experience among Native Americans but rather as medicine for religious and healing purposes.

Limitations include the cross-sectional design of this study, which precludes any causal determinations. The present study also relied on self-reports of hallucinogen use, which could be influenced by under- or over-reporting (e.g., sub-notification). However, the NSDUH incorporated a computer-assisted survey, which should reduce social desirability bias and underreporting among participants given the nature of the sensitive data being collected. Nevertheless, the possibility that some potential participants refused to participate given the sensitive topic could mean that prevalence rates are higher than the estimates presented in this report. Furthermore, some of the smaller sample sizes may reflect some bias in prevalence and trend estimates. Lastly, a small subgroup of the US population resides in prison, hospital or military institutions and thus was not represented in this sample. Despite these limitations, our results contribute to the epidemiological literature and suggest that the population of individuals using hallucinogens is becoming increasingly racial/ethnically diverse as public interest in the potential therapeutic applications of these substances heightens.

## Data Availability Statement

Publicly available datasets were analyzed in this study. This data can be found at: Data for each year, respectively, is available from the following URLs: ^*^ 2015: https://www.datafiles.samhsa.gov/dataset/national-survey-drug-use-and-health-2015-nsduh-2015-ds0001, 2016: https://www.datafiles.samhsa.gov/dataset/national-survey-drug-use-and-health-2016-nsduh-2016-ds0001, 2017: https://www.datafiles.samhsa.gov/dataset/national-survey-drug-use-and-health-2017-nsduh-2017-ds0001, 2018: https://www.datafiles.samhsa.gov/dataset/national-survey-drug-use-and-health-2018-nsduh-2018-ds0001, 2019: https://www.datafiles.samhsa.gov/dataset/national-survey-drug-use-and-health-2019-nsduh-2019-ds0001.

## Ethics Statement

Ethical approval was not provided for this study on human participants because this study was determined exempt by the Iowa State University Institutional Review Board. Written informed consent from the participants' legal guardian/next of kin was not required to participate in this study in accordance with the national legislation and the institutional requirements.

## Author Contributions

AD and BA were responsible for conceptualization and design of the study. BA, AD, YX, and CS were responsible for data analyses and initial interpretation of findings. GA-L and MW contributed to interpretation of findings. All authors contributed to writing and editing of the manuscript.

## Funding

AD was supported by private philanthropic funding from Tim Ferriss, Matt Mullenweg, Craig Nerenberg, Blake Mycoskie, and the Steven and Alexandra Cohen Foundation. AD was also supported by the Center for Psychedelic Drug Research and Education, funded by anonymous private donors. GA-L was supported by funding from NIH grant T32DA007250. The funding sources were not involved in study conceptualization, analyses, interpretation, conclusions, or decision to publish.

## Conflict of Interest

The authors declare that the research was conducted in the absence of any commercial or financial relationships that could be construed as a potential conflict of interest.

## Publisher's Note

All claims expressed in this article are solely those of the authors and do not necessarily represent those of their affiliated organizations, or those of the publisher, the editors and the reviewers. Any product that may be evaluated in this article, or claim that may be made by its manufacturer, is not guaranteed or endorsed by the publisher.
